# Stress phenotyping analysis leveraging autofluorescence image sequences with machine learning

**DOI:** 10.3389/fpls.2024.1353110

**Published:** 2024-04-19

**Authors:** Sruti Das Choudhury, Carmela Rosaria Guadagno, Srinidhi Bashyam, Anastasios Mazis, Brent E. Ewers, Ashok Samal, Tala Awada

**Affiliations:** ^1^ School of Natural Resources, University of Nebraska-Lincoln, Lincoln, NE, United States; ^2^ School of Computing, University of Nebraska-Lincoln, Lincoln, NE, United States; ^3^ Department of Botany, University of Wyoming, Laramie, WY, United States; ^4^ Agricultural Research Division, University of Nebraska-Lincoln, Lincoln, NE, United States

**Keywords:** autofluorescence imaging, stress detection, machine learning-based classifier, high throughput plant phenotyping, drought stress, genotypic variation, *Brassica rapa*

## Abstract

**Background:**

Autofluorescence-based imaging has the potential to non-destructively characterize the biochemical and physiological properties of plants regulated by genotypes using optical properties of the tissue. A comparative study of stress tolerant and stress susceptible genotypes of *Brassica rapa* with respect to newly introduced stress-based phenotypes using machine learning techniques will contribute to the significant advancement of autofluorescence-based plant phenotyping research.

**Methods:**

Autofluorescence spectral images have been used to design a stress detection classifier with two classes, stressed and non-stressed, using machine learning algorithms. The benchmark dataset consisted of time-series image sequences from three *Brassica rapa* genotypes (*CC*, *R500*, and *VT*), extreme in their morphological and physiological traits captured at the high-throughput plant phenotyping facility at the University of Nebraska-Lincoln, USA. We developed a set of machine learning-based classification models to detect the percentage of stressed tissue derived from plant images and identified the best classifier. From the analysis of the autofluorescence images, two novel stress-based image phenotypes were computed to determine the temporal variation in stressed tissue under progressive drought across different genotypes, i.e., the average percentage stress and the moving average percentage stress.

**Results:**

The study demonstrated that both the computed phenotypes consistently discriminated against stressed *versus* non-stressed tissue, with oilseed type (*R500*) being less prone to drought stress relative to the other two *Brassica rapa* genotypes (*CC* and *VT*).

**Conclusion:**

Autofluorescence signals from the 365/400 nm excitation/emission combination were able to segregate genotypic variation during a progressive drought treatment under a controlled greenhouse environment, allowing for the exploration of other meaningful phenotypes using autofluorescence image sequences with significance in the context of plant science.

## 1 Introduction

The development of novel genotypes and implementation of sustainable climate smart practices for resilient agroecosystems are crucial for mitigating the effect of climate change (Climate Change, 2022). Efforts to better understand the complex interactions between genotypes and their natural and managed environment are also relevant for enhancing plant selection and breeding practices and implementing precision nature and technology-based climate-smart solutions that are aimed to enhance productivity, efficiency, sustainability, and resilience of the agroecosystems (Granier et al., 2006; Reynolds et al., 2019; Yang et al., 2020; Fu and Jiang, 2022; Güney et al., 2023). Despite genetic and molecular advances in plant research, rapid and accurate quantification of expressed plant phenotypes remains a bottleneck (Evans and Lawson, 2020). High-throughput plant phenotyping (HTPP) has been increasingly used in agricultural research to inform the selection of desirable plant traits for breeding purposes and to study their interaction with the environment (Yang et al., 2020). Image-based HTPP platforms facilitate the quantification of holistic (*i.e.*, whole plant) and component (*i.e.*, cell to organ level) phenotypes by repeated non-destructive measurements of a large number of individuals in a relatively short time (Das Choudhury et al., 2018; Das Choudhury et al, 2019). The use of machine learning algorithms and artificial neural networks has made the large available heterogeneous images from HTPP platforms computationally tractable (Goodfellow et al., 2016; Goodfellow et al, 2005; Singh et al., 2016). Moreover, the availability and accessibility to different imaging sensors and the opportunity for a combined integrated analytical approach have led to the development of novel applications for image-based HTPP (Pasala and Pandey, 2020; Yang et al., 2020). One such line of research is the study of plant responses to stresses and the introduction of image-based classifiers for stress detection (Das Choudhury et al., 2023).

**Figure 1:**
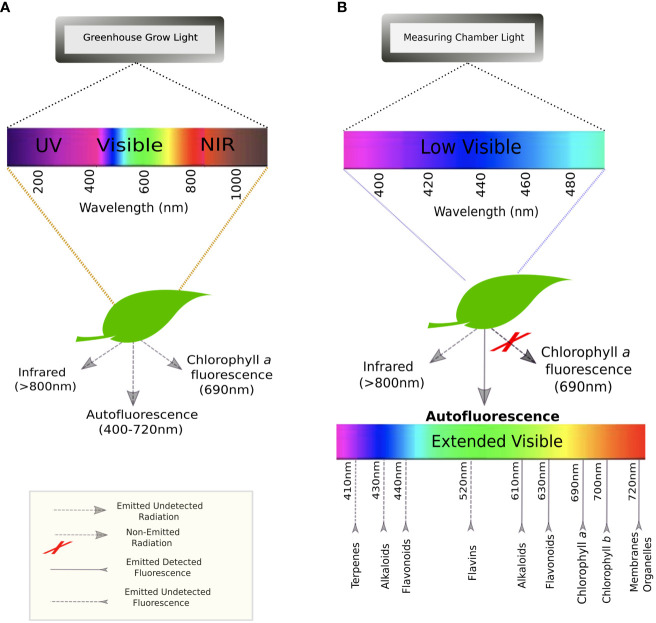
Spectra of excitation and emission for leaves: **(A)** in a greenhouse environment where they are exposed to the entire light spectrum from low UV to the near-infrared; and **(B)** in the autofluorescence measuring chamber at UNL where plants are exposed to low visible/UV light to re-emit an extended visible spectrum.

When plants are exposed to radiation, they can either absorb, transmit, or reflect photons (Vogelmann and Bjorn, 1986). Luminescence refers to the molecules’ capacity to absorb light and then re-emit it at a longer wavelength (*i.e.*, lower energy). This occurs due to the return of electrons from an excited to ground state. If this re-emission happens almost instantaneously, this is known as fluorescence (Valeur and Berberan-Santos, 2011). When plants receive electromagnetic UV or short-wave visible radiation, they emit fluorescence that can be recorded on the extended electromagnetic spectrum of visible light (400-720 nm), peaking at about 440 nm (blue), 520 nm (green), 690 nm (red), and 720 nm (far-red) (Buschmann et al., 2001; Lichtenthaler and Buschmann, 2001) (**Figure 1A**). Chlorophyll *a* fluorescence has been widely studied and used in phenotyping due to its connection to the photosynthetic rate and photosystem II efficiency (Baker, 2008; Murchie and Lawson, 2013). But chlorophyll *a* is not the only fluorophore occurring in plants. Other chlorophylls and their catabolites (*e.g*., porphyrins), as well as lignin, alkaloids, terpenoids, and several phenolic compounds, such as anthocyanins and flavonoids, participate in the emission of (auto)fluorescence (endogenous from plant tissue) (Donaldson, 2020; Barboza da Silva et al., 2021) (**Figure 1B**). Several functions have been attributed to this phenomenon, from visual cue for pollinators to safety valve for absorbed UV radiation (García-Plazaola et al., 2015).

**Figure 2:**
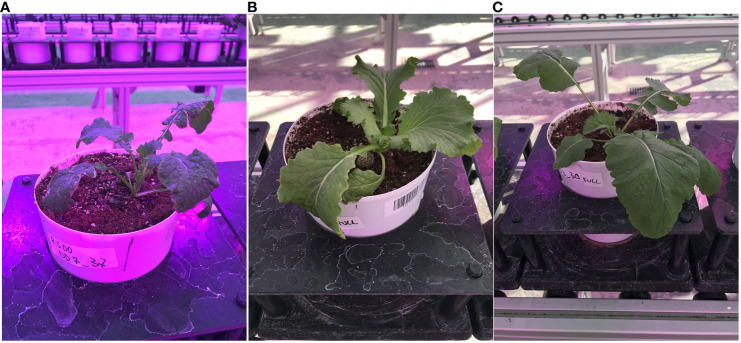
Lateral view of the three *Brassica rapa* genotypes used in the study: **(A)** the oilseed, *R500*; **(B)** the Chinese Cabbage, *CC*; and **(C)** the Vegetable Turnip, *VT*.

In this research, we used high-throughput imaging, computer vision-based algorithms, and machine learning to detect and analyze changes in autofluorescence during a progressive drought experiment in the species *Brassica rapa*, a globally cultivated crop with extreme intraspecific diversity, making it an excellent test case for image analysis. The accuracy of HTPP-derived data was recently tested for an inter-varietal substitution population of a close relative of *B. rapa*, rapeseed (Li et al., 2020). We specifically analyzed the power of HTPP–derived autofluorescence data to detect leaf tissue impacted by drought stress and evaluate if the autofluorescence images are able to pick up any genotypic variation in drought resistance over time. We examined the response of three genotypes of *B. rapa* (a Chinese Cabbage*, CC*, an oilseed variety, *R500*; and a vegetable turnip type, *VT)*, characterized by large variation in their life cycle, morphological and physiological traits (**Figure 2**) (Edwards et al., 2011; Edwards et al., 2012; Edwards et al., 2016; Pleban et al., 2020).

To test the use of HTPP–derived autofluorescence data for drought stress detection in *B. rapa* and to obtain stress phenotypes that computationally separate stressed *vs.* non-stressed leaf tissue, we: 1) pre-processed the images to obtain plant regions, 2) developed and trained a pixel-based algorithm to quantify drought stress from the image sequences, 3) built a novel classifier to discriminate levels of drought stress, and 4) introduced and evaluated two novel stress-based phenotypes, *i.e.*, the average percentage stress and the moving average percentage stress, to highlight possible temporal variation in the drought progression across different genotypes.

## 2 Related works

The use of fluorescence microscopy has significantly improved in recent years to study the biochemical and physiological characteristics of plants (Chacko and Eliceiri, 2019; Donaldson, 2020; Pegg et al., 2021; Zhu, 2022). The two most important auto fluorescent molecules found in plants are chlorophyll (orange/red fluorescence) and lignin (blue/green fluorescence) (Donaldson, 2020). Chlorophyll fluorescence is mainly used to measure the photosynthetic capability of plants, whereas lignin fluorescence is used to evaluate wood phenotyping and assess cell wall porosity. Autofluorescence images have been effectively used to detect the location and degree of lignin deposition during defense response in apple roots to *Pythium ultimum* infection. This study helps in the understanding of the macro-level plant resistance phenotypes regulated by genotypes (Zhu, 2022). An improved understanding of the relationship between a plant’s genotype and its internal anatomical structures may lead to new insights for plant breeding and new ecological and evolutionary dynamics (He et al., 2017). A study conducted by Pegg et al. (2021) on Taxa replicates shows that fluorescence microscopy can be an effective tool for visualizing a plant’s internal anatomical structures with a high degree of precision. Autofluorescence lifetime imaging microscopy has been successfully used by Chacko and Eliceiri, 2019 to measure cellular metabolism in plants.

However, despite the extensive use of autofluorescence in cellular microscopy (Kitin et al., 2020; Pegg et al., 2021), its mechanistic relation to plant metabolism and photosynthetic efficiency has not been fully explored yet (Donaldson, 2020). Since all secondary metabolites responsible for autofluorescence are somewhat influenced by environmental interactions, attempts have been made to use autofluorescence as a stress indicator. Specifically, the impacts of fungal infections on the autofluorescence signal have been studied in grapevine and palm, along with strong allelopathic interactions among pollen cells (Lichtenthaler and Buschmann, 2001; Dreyer et al., 2006; Bellow et al., 2013). Although several HTPP platforms are equipped with cameras to capture autofluorescence, the analysis of these high-throughput data and their possible use in stress detection are just emerging, with pixel-level analysis so far used mostly for thermal and hyperspectral image elaboration (Yang et al., 2020; Li et al., 2021).

The rest of the paper is organized as follows. Section 3 provides materials and methods comprising experimental design, image pre-processing, algorithms for ground truth generation and building classifier for stress detection, and computation of stress-based phenotypes. Section 4 shows analysis results. Finally, Section 5 provides discussions and Section 6 concludes the paper.

## 3 Material and methods

**Figure 3:**
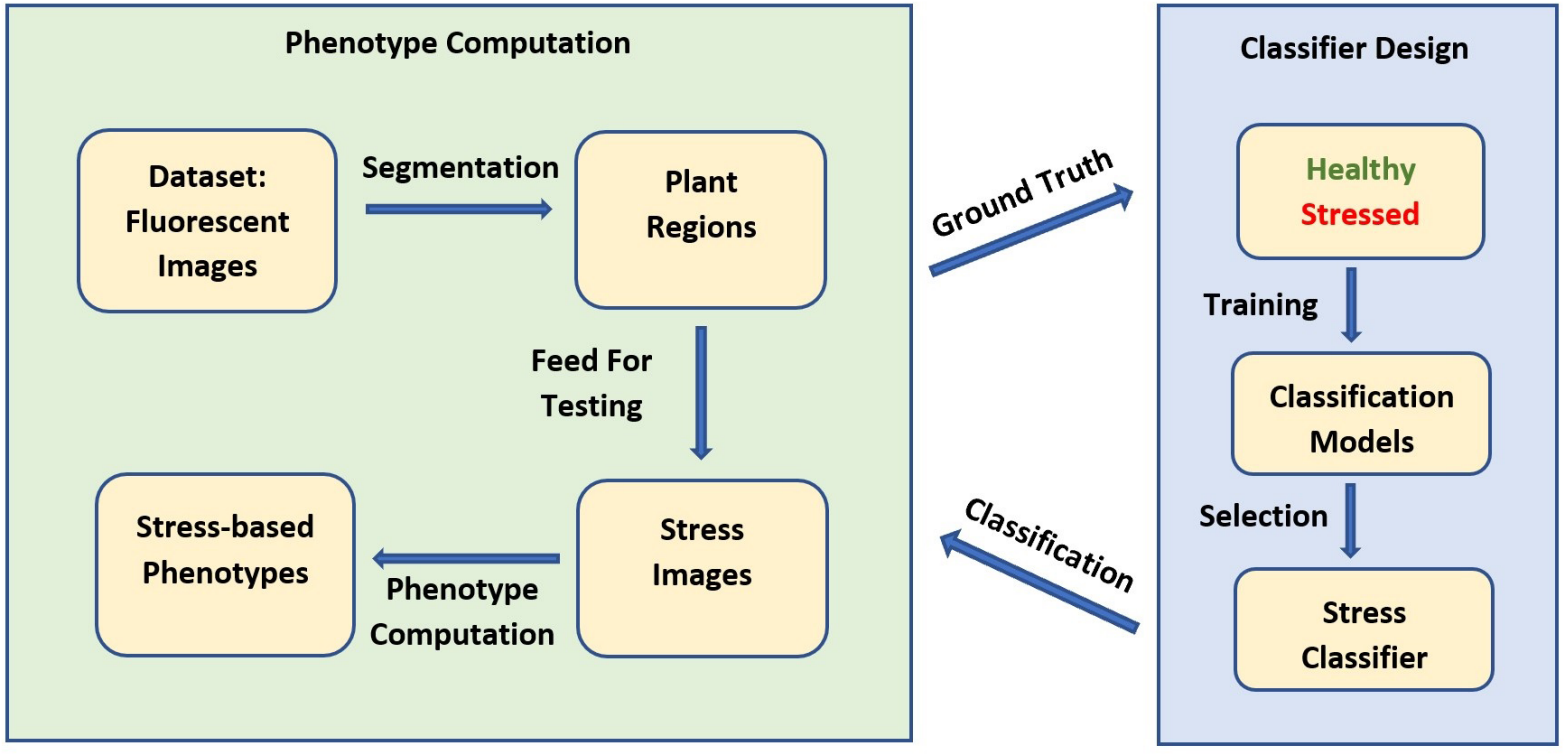
Block diagram for stress detection and stress-based phenotype computation using HTTP-derived autofluorescence image sequences.

The block diagram in **Figure 3** represents the analytical pipeline used in our application. The proposed method consists of two parts: the classifier development for stress detection and the actual computation of phenotypes from autofluorescence images using the classifier. Prior to building the classifier, a dataset is acquired using the HTTP system, consisting of image sequences of *Brassica* plants in both the autofluorescence and the visible spectrum. Both imaging modalities are used to manually create a ground truth dataset to train a stress detection classifier to distinguish stressed pixels from non-stressed pixels. A classifier is selected from a set of single and ensemble classifiers based on their performance on a distinct test set to classify the plant regions into stressed and non-stressed parts. Finally, a set of novel stress-based phenotypes that can be computed from the classified image is proposed.

### 3.1 Experimental design

#### 3.1.1 Plant materials and growing conditions

Plants were grown at the greenhouse equipped with a High Throughput Plant Phenotyping (HTPP) Facility located at the Nebraska Innovation Campus of the University of Nebraska-Lincoln in the fall of 2018. This section provides discussions on selection of genotypes, environmental conditions, and treatment.

*Genotypes:* Three genotypes of *Brassica rapa* that span a wide range of phenotypic and morphometric traits were selected for this experiment: an oilseed variety, *R500*; a vegetable turnip type, *VT*; and a Chinese Cabbage*, CC*, in replicates of at least six plants, with pots randomly placed on the automated conveyor belt of the HTPP platform. Seeds of *VT* and *CC* were obtained from Wageningen UR Center for Genetic Resources (CGN10995 and CGN06867), while seeds for *R500* plants were part of collections bulked at the University of Wyoming in 2013.

*Environmental conditions:* Red/Blue LED lights were on 14/10 day/night photoperiod, 1800 μmol photons m^–2^ s^–1^ maximum photosynthetic photon flux density (PPFD) including the natural light in the greenhouse, temperature was set at 20 – 22°C/18 – 20°C day/night, and relative humidity averaged 45 ± 5%. All seeds were planted in two liters pots filled with a soil mix (Miracle-Gro Moisture Control Potting Mix; 20% v/v, Marysville, OH, and Profile Porous Ceramic Greens Grade; 80% v/v, Buffalo Grove, IL) amended with one teaspoon of Osmocote 18-6-12 fertilizer (Scotts, Marysville, OH) *per* pot.

*Stress treatment:* Until day 7 after germination (DAG), all plants were automatically weighed and watered daily on the HTPP system to maintain soil volumetric water content ~ 35 ± 7% across all genotypes. At 10 DAG, a more consistent progressive drought was obtained by total water withholding on a subset of at least six plants *per* genotype, while the drought response was followed until 30 DAG with a soil water content of 4 ± 2%. To ensure progressive drought for all plants, volumetric soil water content was checked at least once or twice daily (ECH2O; METER Group, Pullman, WA) to adjust the watering regime accordingly. The data analyses did not cover images collected between 0 and 4 DAG since they showed a high noise-to-signal ratio due to the small size of the plants. Moreover, the seedlings did not show any sign of visible stress before the application of drought.

#### 3.1.2 Data acquisition

**Figure 4:**
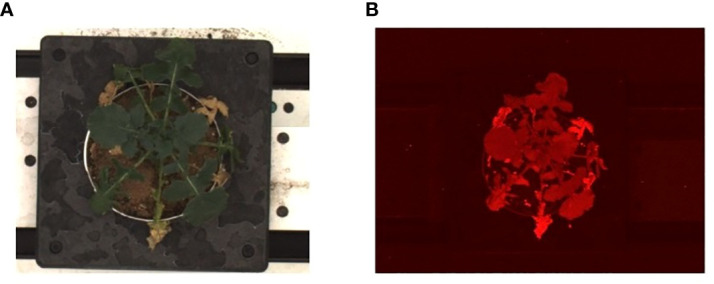
Sample images of the UNL-UW-AFD dataset: **(A)** an image captured with a visible light camera for an *R500* plant at 28DAG; and **(B)** an image captured using the autofluorescence camera of the same individual.

All images were recorded using the High-Throughput Plant Phenotyping Facility (Scanalyzer 3D, LemnaTec Gmbh, Aachen, Germany) at the University of Nebraska-Lincoln. All pots were screened daily starting at 1200p.m., from 7 DAG and for the duration of the experiment. Every imaging period consisted of the movement of pots sequentially on the conveyor belt, first through an adaptation environmental tunnel for 20 minutes (with 800 μmol photons m^–2^ s^–1^ PPFD) and then through four sequential imaging chambers instrumented with different light panels and cameras (Rahaman et al., 2015): Red-Green-Blue (RGB) side view and top view (Prosilica GT6600 29 megapixel camera with a Gigabit Ethernet interface), thermal/infrared side view and top view (Pearleye p-030 LWIR), autofluorescence side view and top view (Basler Scout scA1400-17gm/gc), hyperspectral side view (Headwall Hyperspec Inspector x-vnir) and near-infrared top view (Goldeye p-008 SWIR) (Mazis et al., 2020; Das Choudhury et al., 2023). For this study, we focused on images acquired in the chamber for fluorescence equipped with continuous blue lights (400-450 nm, low wavelength) to excite plant autofluorescence and a camera (resolution: 1390X1038 pixel, 24bit) with apposite filters to record top images between 620-900 nm (**Supplementary Figure S1**). We utilized the top-view RGB images (400-700 nm, resolution 2454 × 2056 pixel, 24 bit) as visual ground-truthing. A total of 3360 images were analyzed during the study to assess drought response between 15 and 30 DAG for the three genotypes of *B. rapa*. For novel algorithm development and systematic evaluation, we built and made publicly available Autofluorescence Dataset collaboratively developed by the University of Nebraska–Lincoln and the University of Wyoming (UNL-UW-AFD) as a benchmark dataset, at https://plantvision.unl.edu/dataset. The dataset consists of 3360 autofluorescence images captured for three genotypes, i.e., *R500*, *CC*, and *VT*. **Figure 4** shows sample images from UNL-UW-AFD dataset. **Figure 4A** shows an image captured with a visible light camera (RGB) for an *R500* plant at 28DAG and **Figure 4B** shows an image captured using the autofluorescence camera of the same individual.

### 3.2 Image pre-processing

**Figure 5:**
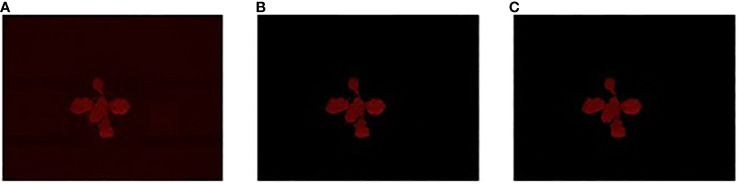
Illustration of the preprocessing steps for the stress phenotyping analysis using a sample image from the UNL-UW-AFD dataset: **(A)** an *R500* individual (14 DAG) is imaged for autofluorescence; **(B)** the extracted foreground image through color-thresholding; and **(C)** foreground image after noise removal.

The pre-processing mainly focused on the segmentation of the autofluorescence images to extract the plant regions. We used color threshold-based segmentation, which extracts the plant from the background based on the color difference. We adopted different color channels (RGB channel and HSV- hue, saturation, and brightness - channel) for different plant images. For the plant images taken in the first few days after germination, the HSV channel gives better results as the plant is small and the autofluorescence intensity is very close to the soil. As the plant grows, the RGB channel performs better separation with the red channel to distinguish the plant region and the background. Further processing was needed to remove random noise due to other materials besides the plant, such as soil mixture, pot, *etc*. An illustration of the pre-processing steps is shown in **Figure 5** using a sample image from the dataset, an *R500* individual at 14 DAG. For each raw autofluorescence image (**Figure 5A**), the foreground was extracted through color-thresholding (**Figure 5B**). Finally, after noise removal, the extracted plant region was used for all subsequent analyses (**Figure 5C**).

### 3.3 Ground truth generation

**Figure 6:**
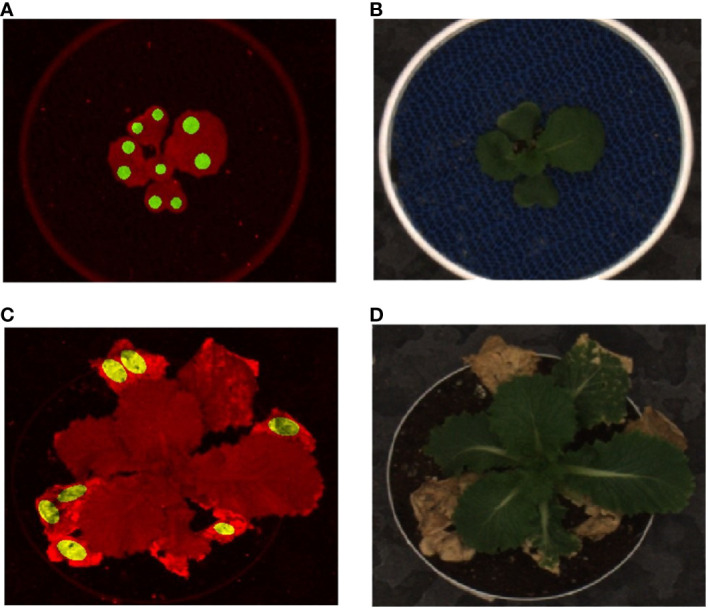
Illustration of ground truth generation: **(A)** autofluorescence image with circular green patches representing non-stressed pixels for a well-watered plant; **(B)** the corresponding RGB image before introduction of stress; **(C)** the fluorescent image with oval shaped yellow patches representing stressed pixels; and **(D)** the RGB image after introduction of drought stress 19DAG. A Chinese cabbage, *CC* genotype of *Brassica rapa* is represented at 14DAG (A&B) and at 28DAG (C&D).

A classifier learns from a set of labeled data that includes samples from the different classes, i.e., ground truth. A subset of segmented images is chosen to be labeled as the ground truth and used for the development of the model and its evaluation. The pixels of the chosen image are labeled as stressed or non-stressed based on their appearance. The non-stressed class denotes the photosynthetically efficient parts of the plant canopy, still green and not affected by drought or browning (Guadagno et al., 2017), while the stressed class denotes drought stressed tissue, which also appeared brown (i.e., dead tissue) in the RGB images. The segmented images before consistent drought stress showed the intensities of autofluorescence pixels in non-stressed regions of the above-ground part of the plants. We extracted such pixels using a number of round-shaped patches on the leaves, slightly away from the larger veins of the leaves (green patches in **Figures 6A,B**). Similarly, the stressed class of pixels is contained in the dried regions of plant images, which occur after the introduction of drought stress. Using the RGB images as reference, similar oval-shaped patches were manually extracted, representing the stressed class (yellow patches in **Figures 6C, D**. The ground truth consists of the pixel intensities extracted from these stressed and non-stressed regions on leaves from all three genotypes: *R500*, *CC*, and *VT*. **Algorithm 1** outlines the steps to generate the ground truth.

Algorithm 1 **Ground truth generation.**

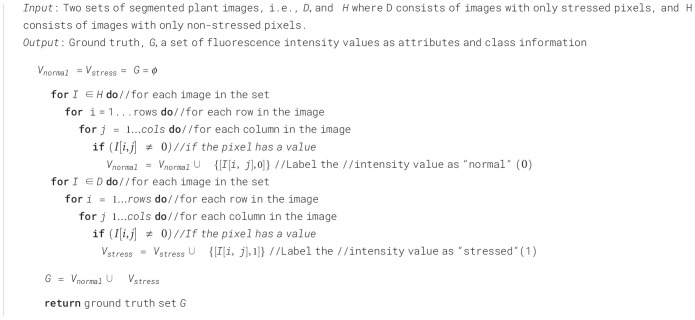

### 3.4 Building a classifier for stress detection

The problem of developing a stress detection classifier can be formulated as follows. Given a set of *m* plants *P* = {*P*_1_, *P*_2_, ...*P_i_*, ...*P_m_*} and each plant *P_i_* represents a list of n fluorescence images {*I_i,_*_1_, *I_i,_*_2_, ...*I_i,j_*, ...*I_i,n_*} where j denotes the imaging date of the plant, along with a $t_{i}$ denoting the day when the stress was introduced to the plant $P_{i}$ we need to develop a classifier C that converts the image $I_{i,j}$ to a stress image $I_{i,j}^{s}$ at the pixel level. If the plant $P_{i}$ is not stressed, the $t_{i}$ will be 0 . The mathematical definition of the classifier is given below:


$$I_{i,j}\left[x,\;y\right]\overset{C}{\to }I_{i,j}^{s}\left[x,\;y\right]\in \left\{0,1\right\}$$

where {0,1} denotes the two classes: 0 denotes the non-stressed class and 1 denotes the stressed class. All mathematical notations for the current study are summarized in **Table 1**.

**Table 1:** Mathematical notations for **Algorithm 1**, **2**.

$P_{i}$	The plant with ID i
$I_{i,j}$	The plant region images of plant at day
$t_{i}$	The day when the stress been introduced
C	The stress detection classifier
T	A set of stress-based plant phenotypes
G	The ground truth dataset
$V_{normal}$	A subset of fluorescence intensity values labelled as normal (non-stressed) parts
$V_{stress}$	A subset of fluorescence intensity values labelled as stress (dry) parts

To separate the non-stressed and the stressed areas of the plant, we utilized multiple machine learning-based classification models (Zhou, 2021) and evaluated their accuracies. These models include Ensemble (Bagged Trees), Neural Network (Medium Neural Network with a fully connected layer of size 25), Support Vector Machines (SVM, Quadratic kernel function), Decision Tree (Medium Tree with a maximum of 20 splits), Logistic Regression, and Naïve Bayes (Gaussian numeric predictor). The classifiers aim to find a boundary between the stressed and non-stressed classes based on the ground truth. **Algorithm 2** is used to build the stress detection classifier.

Algorithm 2 **Building stress detection classifier.**

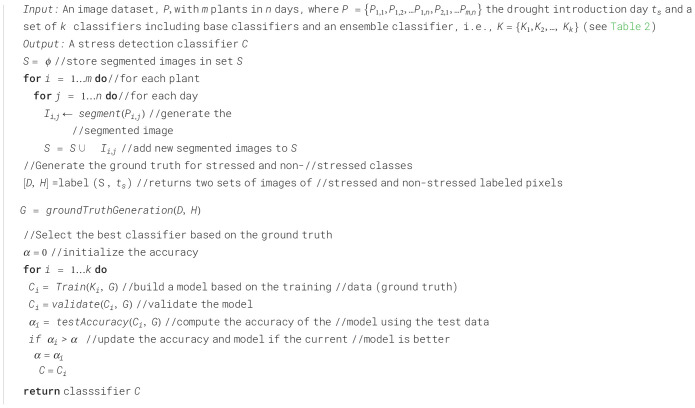

**Table 2:** Comparative performance analysis of classifiers for stress detection based on autofluorescence image analysis.

Machine Learning Classification Model	Validation Accuracy	Test Accuracy
Ensemble	98.30%	99.80%
Neural Network	98.50%	98.70%
SVM	98.50%	98.50%
Decision Tree	98.00%	98.10%
Logistic Regression	97.50%	97.50%
Naive Bayes	96.70%	96.50%

### 3.5 Computation of stress-based phenotypes

**Figure 7:**
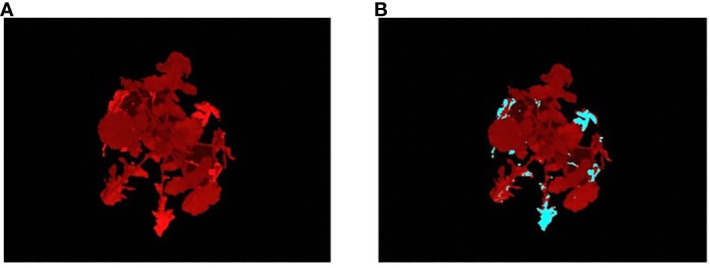
Illustration of the results of classifier: **(A)** an image of a *R500* plant at 28DAG captured using a fluorescent camera; and **(B)** the stress image of the same individual, where the predicted stressed regions of the plant are marked in blue color.

To compute the stress-based phenotypes, the stress pixels from the plant region images are first extracted using the best performing classifier. **Figure 7** shows a sample result using the classifier. The stressed parts in the image are marked in blue, matching the yellow leaf parts in the visible light image, indicating the separation of stressed and non-stressed parts of the plant. The non-stressed parts of the leaves were left at the original color (**Figures 7A, B**).

We propose two novel stress-based plant phenotypes that can be directly computed from the stress images generated after the classification. The phenotypes measure the stress in a plant and the temporal changes over time. They are briefly described below.

Average Percentage Stress: The average percentage of stress for a given genotype on any given day is computed as the average percentage of stressed tissue, represented as the number of stressed pixels in the plant. The higher the average percentage stress, the more stressed tissue was present across the plant canopy. Since stress response to drought is highly variable across individuals, results are presented as the average percentage stress values of all the plants in each genotype.

Moving Average Percentage Stress: The moving average percentage stress for a given plant on any given day is computed by averaging the mean of the average percentage stress values for that plant for the past n ways (temporal window size), including the value on the given day, where n can be determined experimentally. This phenotype is computed from the time series of a stress phenotype and captures the temporal trends of stress induction.

## 4 Results

In this section, we demonstrate the results of the comparative performance analysis of the stress detection classifiers to select the best performing model for our application and the stress-based phenotypes that show genotypic variation during drought stress propagation.

### 4.1 Classifier for stress detection

We examined the performance of six classifiers using a validation set and a separate test set. Their performance is summarized in **Table 2**. All classifiers performed well, with test accuracy ranging from 96.5% (Naïve Bayes) to 99.8% (Ensemble). Since the ensemble model achieved the highest accuracy, it was used to classify the images for phenotype computation.

### 4.2 Genotypic variation for stress-based phenotypes

We attempted to identify genetic variation within *B. rapa* types under drought using autofluorescence imaging. Specifically, we recorded images at midday on three extreme genotypes of *B. rapa* (a Chinese cabbage (*CC*); a vegetable turnip (*VT*); and an oilseed, *R500*) (**Figure 1**).

**Figure 8:**
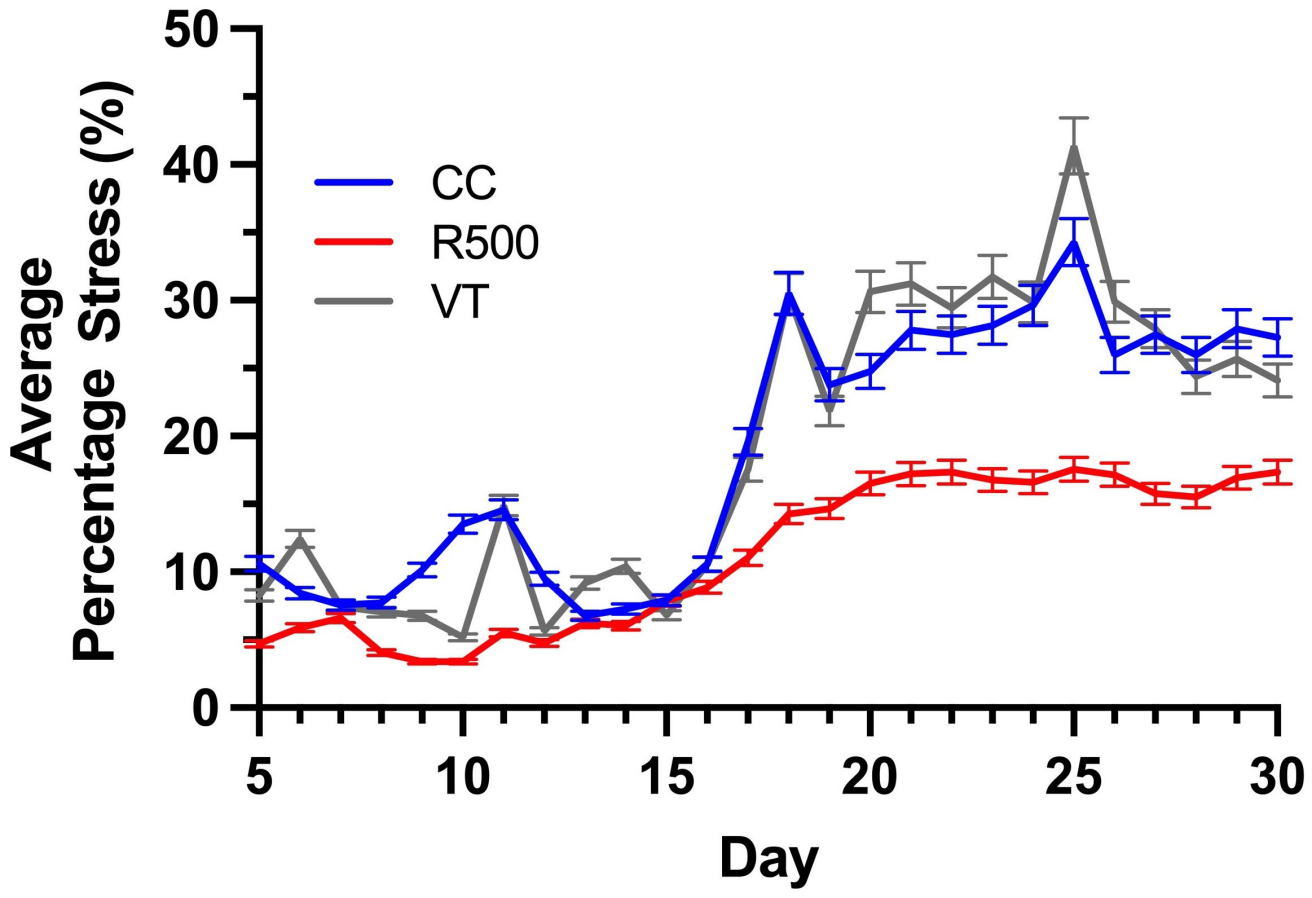
Genotypic variation for the average percentage stress over the time of drought for three *Brassica rapa* genotypes, *R500* (gray), *CC* (blue), and *VT* (orange). The bars represent standard error for at least five replicate individual plants analyzed to compute the phenotype.

We evaluated the average percentage of stressed tissue for each replicate plant and the moving average over the course of a progressive drought to assess possible temporal variations in the genotype stress behavior (**Figures 8** **,** **9**). By studying these two computed imaging phenotypes, our imaging approach permitted the detection of significant differences across genotypes, consistent with previous findings for the same genotypes under progressive drought (Guadagno and Ewers, 2020). In particular, for *R500*, the average percentage of stress tissue was consistently lower than the other two genotypes throughout the duration of the analysis, between 5 and 30 DAG (**Figure 8**). This difference became significant (*p*< 0.001) after day 20 when *R500* started to plateau at 15 ± 1% while the other two genotypes showed a further increase in tissue damage. *VT* and *CC* showed a very similar response to the drought treatment for tissue damage, with only a few significant differences on particular days, such as days 9, 10, 20, and 25 (*p*< 0.005) (**Figure 8**). The turnip type is characterized by very broad leaves, high presence of thorns, and a relative water content higher than *R500* which explained the greater visible impact of drought on the above-ground tissue (Guadagno et al., 2021). When compared to cabbage, *R500* presents more but smaller and thinner leaves than the cabbage genotype, allowing for a more moderate impact of drought on the oilseed genotype.

**Figure 9:**
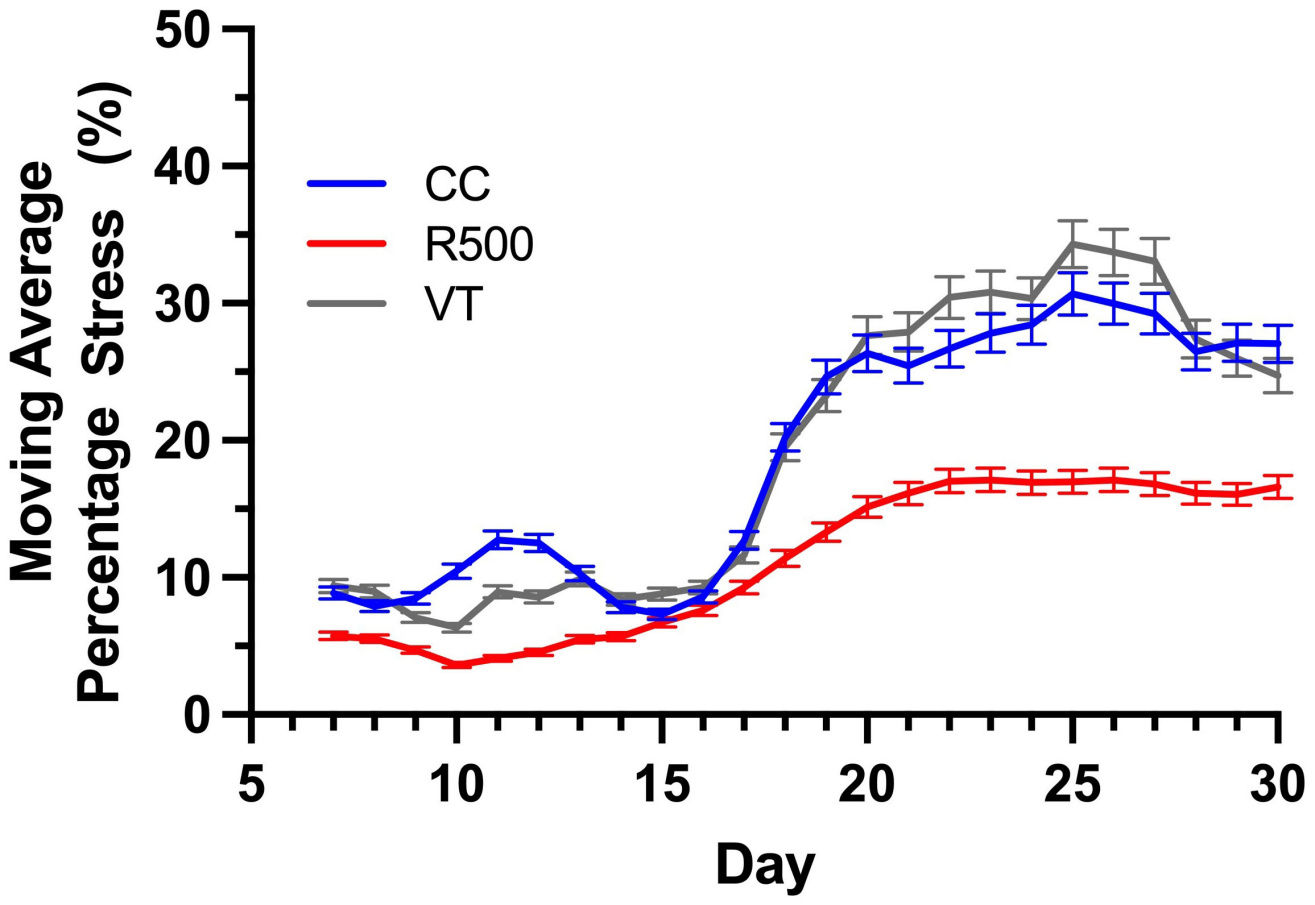
Genotypic variation for the moving average percentage stress over the time of drought for three *Brassica rapa* genotypes, *R500* (gray), *CC* (blue), and *VT* (orange). At least five replicate individual plants were analyzed to compute the phenotype.

The presented HTPP experiment applied a moderate level of drought between days 7 and 30, and our outcomes for *R500* align with previous results on this genotype for different durations and strengths of drought (Greenham et al., 2017; Guadagno et al., 2021). To better highlight the difference between genotypes, we computed the moving average of the tissue damage to determine the direction of drought and trend for each genotype (**Figure 9**).

Irrespective of the genotype, a significant increase in tissue stress was observed with increased drought after 15 DAG (**Figure 8**). Specifically, the increased autofluorescence intensity, as expressed as the number of pixels *per* leaf area, is indicative of energy re-emission from an array of different metabolites (**Figure 1B**). In the non-stressed tissue this autofluorescence is masked by the presence of the chlorophylls that do not re-emit at the same wavelength (Buschmann et al., 2001; Lichtenthaler and Buschmann, 2001). The drought-induced depletion in chlorophylls – underlined by the visual browning of the leaves (**Figure 6D**) – allowed for autofluorescence quantification across all genotypes of *B. rapa*, confirming that imaging autofluorescence can play a primary role as an HTPP method to identify and quantify tissue damage due to environmental stresses.

## 5 Discussion

Characterizing genotypic variation to identify and predict adaptive stress responses is crucial for both agricultural and breeding purposes (Sircar and Parekh 2015; Debieu et al., 2018; Ray et al., 2019). Within a species, genotypes may respond differently to environmental stresses due to different alleles or an intrinsic varying allelic sensitivity at causal loci (Edwards et al., 2011; Matsui and Ehrenreich, 2016). As a consequence, the ability to screen across genotypes under progressive stress can explain genetic effects that may not be related to the trait of interest, ultimately impacting current breeding and management practices (Kumar et al., 2015; Cooper et al., 2019). Autofluorescence is the result of energy from several metabolites when activated by short wave UV and visible radiation (**Figure 1**). To explore the possible mechanistic significance of this plant characteristic re-emission, image sequences from an HTPP system captured by using a fluorescent camera were tested for possible detection of genotypic variation of phenotypic traits across three extreme genotypes in the species *B. rapa* (**Figure 2**). A novel pipeline for the utilization of autofluorescence images using computer vision and machine learning techniques was built for the classifier design and the phenotype computation (**Figure 3**). The paper includes two novel algorithms for ground truth generation (**Algorithm 1**) and building stress detection classifier (**Algorithm 2**) to provide step by step guidance for the concise representation of this method (**Figures 4** **–** **7**). Non-destructive image-based measurements of phenotypic traits enabled the detection of significant (*p*< 0.01) differences in drought stress induction between *R500*, an oil seed type, and both *VT*, and *CC*, a vegetable turnip, and Chinese cabbage, respectively (**Figures 8** **,** **9**).

Localized browning of the leaf tissue caused by stress is not always indicative of an unrecoverable *status* for the plant but can be used to assess and mitigate drought and other environmental variables. The high-throughput quantification of stressed tissue *via* autofluorescence images has the potential to inform the thresholds of recovery after biotic and abiotic stresses. These thresholds are species- if not genotype-specific, and future implementations of the stress detection algorithms will focus on implementing the power of the predictions across plant species beyond *B. rapa* and for other environmental stresses toward a unified pipeline for image stress detection. These results open an exciting prospect for using autofluorescence in agricultural phenomics (Kumar et al., 2015; Yang et al, 2020).

## 6 Conclusions

Autofluorescence imagery reveals genotypic differences and allows to extract biophysical properties of a plant under a given environmental condition contributing to the understanding of a plant’s vigor. In this paper, autofluorescence image sequences obtained from an HTPP platform were used to quantify the drought stress response of three *B. rapa* genotypes (*VT*, *CC*, and *R500*). We developed a novel stress detection algorithm and built a stress detection classifier based on the ensemble machine learning model to classify the plants’ tissue as either stressed or non-stressed. We introduced two phenotypes, i.e., average percentage stress and the moving average percentage stress. Experimental evaluation shows that autofluorescence based phenotypes are influenced by genotypic variation during drought stress propagation over time. Overall, the *VT* and *CC* genotypes showed a similar response to the drought treatment for tissue damage, with a sharper increase in autofluorescence during the period of consideration compared to *R500*. The stress phenotypes indicated that the *VT* and *CC* genotypes are more susceptible to drought stress compared to the *R500* genotype. A benchmark dataset, i.e., UNL-UW-AFD, has been released to facilitate the development of new algorithms and performance evaluation of the competing methods.

## Data availability statement

The original contributions presented in the study are included in the article/**Supplementary Material.** Further inquiries can be directed to the corresponding author. The dataset (UNL-UW-AFD) used in the study can be freely downloaded from https://plantvision.unl.edu/dataset.

## Author contributions

SC: Conceptualization, Formal analysis, Funding acquisition, Methodology, Project administration, Supervision, Writing – original draft, Writing – review & editing, Investigation. CG: Methodology, Resources, Writing – original draft, Writing – review & editing, Supervision. SB: Formal analysis, Methodology, Software, Writing – original draft, Data curation. AM: Data curation, Formal analysis, Writing – review & editing. BE: Resources, Writing – review & editing. AS: Investigation, Project administration, Supervision, Writing – review & editing. TA: Funding acquisition, Writing – review & editing.

## Supplementary material

Supplementary Figure 1
**(A)** The view of the HTPP facility hosting the plants on the conveyor belts; **(B)** sample plants entering the autofluorescence chamber of the HTPP facility; and **(B)** the top panel of low visible/UV lights for autofluorescence induction inside the chamber.
