# Supplementary figures and images for: Stress phenotyping analysis leveraging autofluorescence image sequences with machine learning
Source: Front Plant Sci. 2024 Apr 19;15:1353110. doi: 10.3389/fpls.2024.1353110 (PMC11066247; doi:10.3389/fpls.2024.1353110)

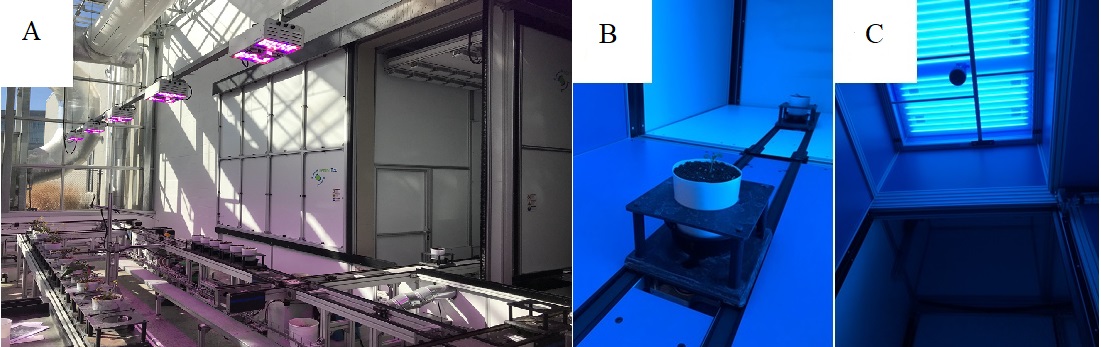

Supplement: Supplementary Figure 1 — (A) The view of the HTPP facility hosting the plants on the conveyor belts; (B) sample plants entering the autofluorescence chamber of the HTPP facility; and (B) the top panel of low visible/UV lights for autofluorescence induction inside the chamber. [file Image_1.jpeg]
